# Online interpersonal trust and online altruistic behavior in college students: the chain mediating role of moral identity and online social support

**DOI:** 10.3389/fpsyg.2024.1452066

**Published:** 2024-09-12

**Authors:** Daokai Sun, Yingying Lin, Chuanjing Liao, Lili Pan

**Affiliations:** ^1^Mental Health Education Center, Wenzhou University, Wenzhou, China; ^2^Chinese Culture College, Wenzhou-Kean University, Wenzhou, China; ^3^School of Education, Wenzhou University, Wenzhou, China; ^4^College of Mathematics and Physics, Wenzhou University, Wenzhou, China

**Keywords:** online interpersonal trust, moral identity, online social support, online altruistic behavior, college students

## Abstract

**Background:**

The prevalence of online altruistic behaviors among the college students has attracted widespread attention. However, the factors influencing this are still unclear. The objective of this study was to explore the relationship and mechanism of online interpersonal trust, moral identity, online social support and online altruistic behavior among college students.

**Methods:**

The survey was conducted among 986 Chinese college students using the Interpersonal Trust Scale for the Internet, Moral Identity Scale, The Internet Social Support Questionnaire for College Students and The Internet Altruistic Behavior Questionnaire for College Students.

**Results:**

Moral identity, online social support, online interpersonal trust and online altruistic behavior were significantly positively correlated (*r* = 0.09–0.39, *p* < 0.01). Online social support plays a partial mediating role in the relation between online interpersonal trust and online altruistic behavior, accounting for 33.76% of the total effect, while moral identity and online social support play a chain mediating role in the relation between online interpersonal trust and online altruistic behavior, accounting for 2.23% of the total effect.

**Conclusion:**

Online interpersonal trust not only directly affects college students’ online altruistic behavior, but also indirectly influences it through moral identity and online social support.

## 1 Introduction

The rapid popularization of the Internet and social media has prompted many behaviors and phenomena derived from college students’ online interactions. College students engage in beneficial behaviors such as information sharing, interactive learning, job search tips, donations and announcements on virtual communities or networks, and more and more researchers have begun to pay attention to the above altruistic behaviors (Guo and Kang, 2015). Online altruistic behavior refers to an individual’s behavior of voluntarily helping others in a virtual online environment, which is a kind of behavior that benefits others (Zheng et al., 2023). It has been shown that online altruistic behavior holds significant value for the helper, the recipient, and society as a whole (Amichai-Hamburger, 2008; Zhang et al., 2024). It can guide young college students to use the internet correctly and responsibly, enhancing their subjective well-being (Zheng et al., 2018; Stehr, 2023) and self-consistency (Luo et al., 2021). Additionally, online altruistic behavior is closely related to various positive factors, such as healthy interpersonal relationships (Liu et al., 2014; Zheng and Zhao, 2015). Therefore, an in-depth study of the inner psychological mechanism of college students’ altruistic behavior in the network environment is of great value and significance for enriching and developing its related theories, as well as providing guidance for practice.

Trust is a complex concept, with its definition and understanding varying across different disciplines. Online environments offer new possibilities for the emergence of trust, but they also bring challenges (Tagliaferri, 2023). Online interpersonal trust, refers to the general expectation that an individual has on the reliability of the others when interacting with them in cyberspace based on their words, promises, and written or verbal statements (Zhao et al., 2013). Online interpersonal trust is a key factor in promoting the development of online interpersonal interactions, which can enhance the efficiency of network cooperation and the depth of knowledge sharing, and is of great significance to the harmonious, stable and sustainable cyberspace (Sun et al., 2015). Social cognitive theory states that an individual’s perception and understanding of the surrounding environment will influence his or her choice of subsequent behavior (Bandura and Walters, 1977). Relevant empirical findings indicate that there is a positive correlation between the level of online interpersonal trust perceived by an individual and the propensity for online altruistic behavior, i.e., when an individual perceives higher interpersonal trust in an network environment, he or she is more likely to exhibit online altruistic behavior (Zhao and Zhang, 2013). On the contrary, those with low levels of online interpersonal trust will have negative evaluations of others and the environment, and will be less likely to engage in online altruistic behavior. Accordingly, the study proposes Hypothesis 1: Online interpersonal trust is positively correlated with online altruistic behavior.

In a study published in 2011, Johnston and Krettenauer noted that adolescents with a strong sense of moral identity were more engaged in pro-social behaviors, such as helping residents in their neighborhoods (Krettenauer and Johnston, 2011). Social identity theory states that individuals tend to associate themselves with specific groups and derive a sense of identity from the groups. Aquino and Reed define moral identity as a self-concept centered on a specific set of moral traits (Aquino and Reed, 2002). Individuals with high moral identity are more capable of moral self-regulation, facilitating the occurrence of moral actions. Research has indicated that high moral identity can promote individuals’ prosocial behavior (Zeng et al., 2020) and inhibit individuals from engaging in immoral behaviors, such as cyber-deviant behavior and aggressive actions (Yang et al., 2018; Zeng and Xue, 2022). In networks, an individual’s trust in a specific group may lead to moral identification with that group (Rest, 1986). Moral development theory suggests that when it comes to moral identity, individuals change with their developmental stage. Individuals’ moral identity on the Internet may be influenced by their surroundings and social groups, especially trusted groups (Krohn, 2010). Accordingly, the study proposes Hypothesis 2: Moral identity plays a mediating role between online interpersonal trust and online altruistic behavior.

Online social support refers to the sense of belonging and identification to one’s environment and others that an individual experiences through emotional, informational, and material exchanges and interactions in the virtual online world (Liang and Wei, 2008). The characteristics of convenience, disinhibition, high self-disclosure, and high intimacy in online interactions make the social support perceived through the network significantly different from that in the real world (Mickelson, 2014). Individuals with higher levels of trust are more inclined to exhibit trusting behaviors in the same environment (Liu and Rau, 2012), have a stronger ability to perceive and utilize social support, have a more positive view of the environment and others, and support and promote the manifestation and practice of altruistic behaviors. Access to social support in the network enhances an individual’s sense of belonging in cyberspace and prompts a more positive view of the online environment, which may in turn drive more online altruistic behaviors. According to empirical research, the more individuals tend to display more of their strengths while exhibiting less hostile and aggressive behaviors, the more social support they receive in the network (Scarpa and Haden, 2006). Accordingly, the study proposes Hypothesis 3: online social support plays a mediating role between online interpersonal trust and online altruistic behavior.

**Figure 1:**
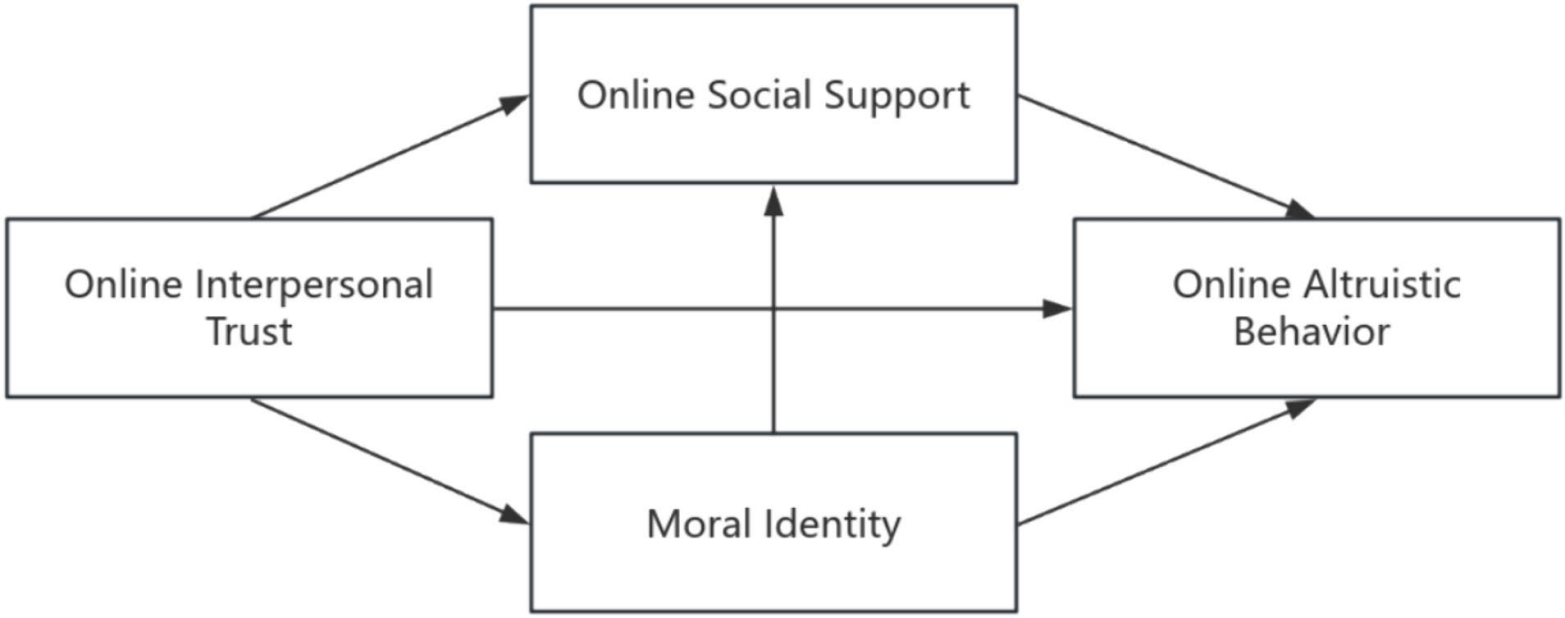
Hypothetical model.

Individuals building trusting relationships in networks may shape their identification and internalization of the moral code of a particular group or network environment, and they may be more likely to identify with the values and moral code of the group when they feel trust from others. This identification may make the individual more willing to follow the moral code endorsed by the group, which in turn influences his or her behavior. Through the internalization of a particular moral identity, an individual may gain a sense of identification and social support for the group. Individuals may be recognized and supported by other members of the group because of their moral identification with the group. Omoto et al. (2002) proposed that social support from organizations, as outlined in their volunteer process model, can foster individuals’ prosocial motivation, which may, in turn, increase and sustain their altruistic behavior. This suggests that social support can stimulate altruistic behaviors. When individuals feel recognized and supported by social groups, they are more likely to exhibit altruistic behavior on online platforms as a way of giving back and contributing to these social groups. In summary, when individuals establish trust in online environments, it shapes their identification with moral standards. This identification may lead to a heightened perception of social support, and further stimulate the display of online altruistic behavior, thereby forming a chain of mediating pathways. Accordingly, this study proposes Hypothesis 4: Moral identity and online social support play a chained mediating role between online interpersonal trust and online altruistic behavior. The chain mediation model is shown in Figure 1.

## 2 Methodology and participants

### 2.1 Subjects

This study adopts the method of sampling survey, takes the class as the unit, selects the undergraduates from six universities in Zhejiang Province, Shandong Province and Heilongjiang Province as the subjects, and distributes 200 questionnaires to each university with a total of 1,416 copies. After collection, 986 valid questionnaires (69.63%) were obtained by excluding those with invalid responses, too short or too long response time (response time is less than 180 s or more than 900 s), 10 consecutive identical options and high homogeneity. The detailed distribution is shown in Table 1.

**Table 1:** Sample distribution of respondents.

	Genders		Single child or not		Grade			
	Male	Female	Yes	No	First grade	Second grade	Third grade	Fourth grade
Number	283	703	300	686	357	220	191	218
Proportion	28.7%	71.3%	30.4%	69.6%	36.2%	22.3%	19.4%	22.1%

### 2.2 Research tools

#### 2.2.1 The interpersonal Trust Scale for the internet (ITSI)

This study used the Interpersonal Trust Scale developed by Ding and Shen (2005). The scale adopts a 5-point scale, ranging from 1 for “completely inconsistent” to 5 for “completely consistent.” The higher the score, the higher the level of online interpersonal trust. The Cronbach’s α coefficient for this scale in this study was 0.82.

#### 2.2.2 Moral Identity Scale (MIS)

The scale used in this study was co-developed by Aquino and Reed (2002) and later translated and localized by Wan and Yang (2008). The scale consists of 16 questions that are categorized into two dimensions: implicit moral cognition and explicit moral cognition. Seven questions (numbered 1, 2, 5, 8, 10, 11, 12, 13) of the scale were specifically designed to assess implicit moral cognition, while the other nine questions (numbered 3, 4, 6, 7, 9, 14, 15, 16) were used to measure explicit moral cognition. The Cronbach’s α coefficient for this scale in this test was 0.96.

#### 2.2.3 The Internet Social Support Questionnaire for College Students (ISS)

In this study, the Internet Social Support Questionnaire for College Students developed by Liang and Wei (2008) was used to measure the level of social support received by individuals in online interactions. The scale was divided into four dimensions, namely emotional support, information support, instrumental support, and social membership support, with a total of 23 items. The higher the score derived from the scale, the more support an individual feels in online socialization. The Cronbach’s α coefficient of the scale in this study was 0.94.

#### 2.2.4 The Internet Altruistic Behavior Questionnaire for College Students (IAB)

This study used the Internet Altruistic Behavior Scale for College Students developed by Zheng et al. (2011), which consists of 26 items with four dimensions: online support, online guidance, online sharing, and online reminders. For each item in the scale, participants were asked to rate their behavior on a four-point scale from “never” to “always,” corresponding to 1, 2, 3, and 4 points, respectively. The Cronbach’s α coefficient for the questionnaire in this study was 0.97.

### 2.3 Statistical analysis

The data analysis for the study was conducted using IBM SPSS 23.0 and the PROCESS V3.3 plug-in, which were chosen for their robust analytical capabilities, compatibility, and the plug-in’s mediation and moderation analysis features. The study used the Model 6 of PROCESS to test the hypothesis and investigate the mediating effect. In PROCESS, a Bootstrap sample of 5,000 was selected, and 95% confidence intervals (CI) were calculated.

## 3 Results and analysis

### 3.1 Common method bias control and test

Based on the fact that the research data were collected via online questionnaires, the results will be affected by common method bias. Therefore, Harman one-way factor analysis was used to carry out statistical tests for common method bias (Xiong et al., 2012). The results showed that a total of 10 factors were greater than 1 when unrotated, explaining a total of 66.44% of the variance, and the first factor explained 26.83% of the variance, which is less than the previous recommended standard: 40%. Therefore, there is no serious common method bias in this study.

### 3.2 Descriptive statistics and correlation analysis between the main variables

Through Pearson correlation analysis, it was found that the four variables of online interpersonal trust, moral identity, online social support and online altruistic behavior were significantly correlated with each other. The correlations, means and standard deviations of the variables are shown in Table 2.

**Table 2:** Descriptive results and correlation analysis of variables (*r*).

Variables	*M ± SD*	1. Online interpersonal trust	2. Moral identity	3. Online social support	4. Online altruistic behavior
1	2.75 ± 0.54	1			
2	3.90 ± 0.74	0.09**	1		
3	3.52 ± 0.64	0.36**	0.27**	1	
4	1.95 ± 0.55	0.31**	0.17**	0.39**	1

*p < 0.05, **p < 0.01.

### 3.3 The mediating role of moral identity and online social support between online interpersonal trust and online altruistic behavior

After the correlation analysis, it was found that there was a significant level of correlation between online interpersonal trust and college students’ moral identity, online social support, and online altruistic behavior, and based on the results of the analysis, the multiple mediation model was further developed. Using the PROCESS macro program prepared by Hayes (2013), model 6 was selected under the condition of controlling gender and grade level, and 5,000 samples were taken to analyze the mediating role of moral identity and online social support between online interpersonal trust and online altruistic behavior. They were chosen for their robust analytical capabilities, compatibility, and the plug-in’s mediation and moderation. The regression analysis between variables showed (see Table 3 for details): online interpersonal trust, moral identity and online social support significantly and positively predicted college students’ online altruistic behavior (*β* = 0.190, *p* < 0.01; *β* = 0.066, *p* < 0.05; *β* = 0.305, *p* < 0.01). Online interpersonal trust, moral identity significantly and positively predicted online social support (*β* = 0.338, *p* < 0.01; *β* = 0.245, *p* < 0.01). And online interpersonal trust significantly positively predicted moral identity (*β* = 0.086, *p* < 0.01).

**Table 3:** Regression analysis between variables in model 1.

Regression equation		Overall fit indicator		Significance of standardized regression coefficients		
Dependent variable	Predictor variable	*R*	*R^2^*	*F*	*β*	*t*
Moral identity	Online interpersonal trust	0.086	0.007	7.342	0.086	2.710**
Online social support	Online interpersonal trust	0.434	0.189	114.281	0.338	11.722**
	Moral identity				0.245	8.504**
Online altruistic behavior	Online interpersonal trust	0.434	0.189	76.049	0.190	6.161**
	Moral identity				0.066	2.206*
	Online social support				0.305	9.572**

*p < 0.05, **p < 0.01.

**Figure 2:**
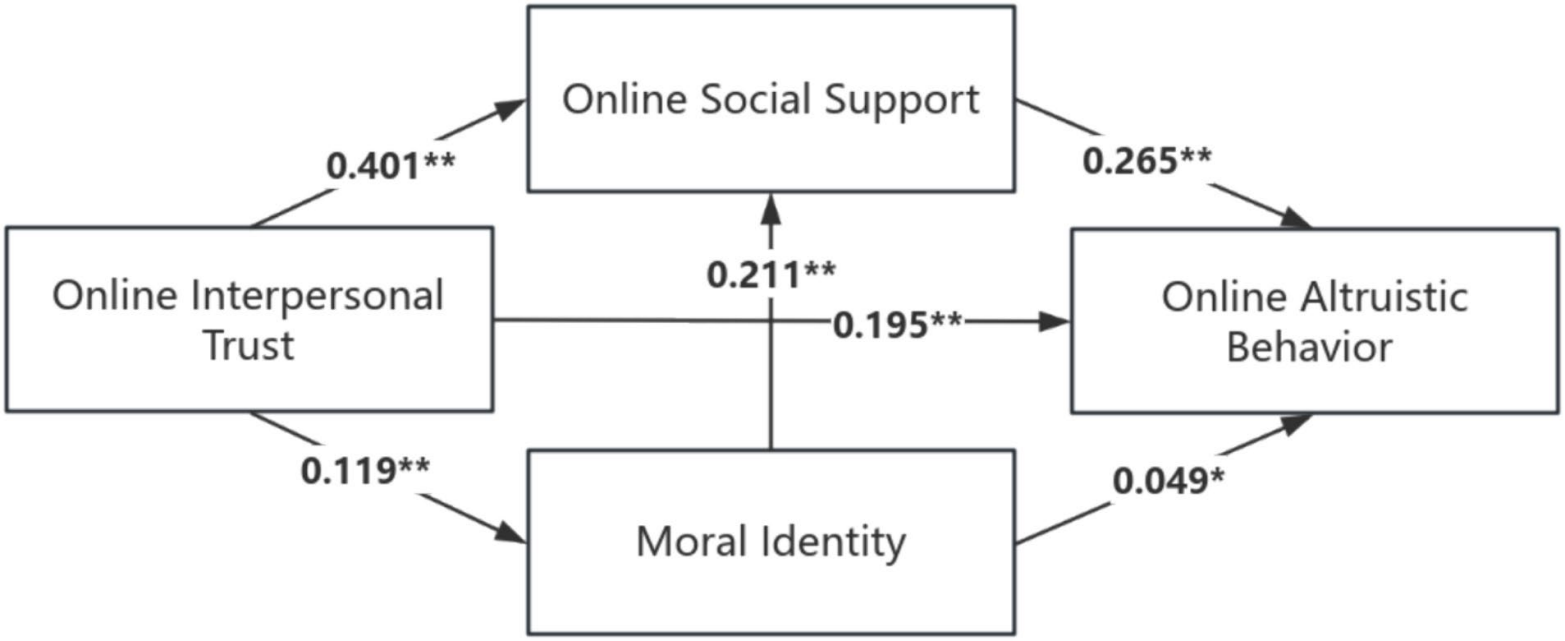
Chain mediation of moral identity and online social support. *p < 0.05, **p < 0.01.

The results of the analysis of the mediating effect show (see Table 4; Figure 2): the mediating effect value of moral identity and online social support between online interpersonal trust and online altruistic behavior is 0.119, and its 95% confidence interval is [0.079, 0.161], which does not contain 0 in the confidence interval, indicating that the mediating effect is significant, accounting for the total effect of life events on sleep quality (0.314) of 37.90%. Specifically, the mediating effect consists of indirect effects generated by three pathways: indirect effect 1 (0.006) through the pathway of online interpersonal trust → moral identity → online altruistic behavior, whose 95% confidence interval is [−0.001, 0.015], and whose confidence interval contains 0, which can indicate that the mediating effect is not significant. The indirect effect 2 (0.110) through the pathway of online interpersonal trust → online social support → online altruistic behavior, with a 95% confidence interval of [0.028, 0.199] and no 0 included in the confidence interval, which can suggest that the mediating effect is significant, accounting for 33.76% of the total effect. Through the pathway of online interpersonal trust → moral identity → online social support → online altruistic behavior produces indirect effect 3 (0.007), whose 95% confidence interval is [0.001, 0.014], and the confidence interval also does not contain 0, which can indicate that the mediating effect is also significant, accounting for 2.23% of the total effect (Figure 2).

**Table 4:** Analysis of mediating effects of moral identity and online social support.

	Effect	BootSE	BootLLCI	BootULCI	Effectiveness as a percentage (%)
Direct effect	0.195	0.031	0.133	0.257	62.10
Path 1	0.006	0.041	−0.001	0.015	1.91
Path 2	0.106	0.019	0.070	0.146	33.76
Path 3	0.007	0.003	0.001	0.014	2.23
Total mediation effect	0.119	0.021	0.079	0.161	37.90

Path 1: online interpersonal trust → moral identity → online altruistic behavior; Path 2: online interpersonal trust → online social support → online altruistic behavior; Path 3: online interpersonal trust → moral identity → online altruistic behavior → online social support → online altruistic behavior.

## 4 Discussion

This study analyzed the relationship between online interpersonal trust and online altruistic behavior and its mechanism of action. The results of the study show that there is a significant positive predictive effect of online interpersonal trust on moral identity, online social support, and online altruistic behavior, i.e., the higher the scores of online interpersonal trust of college students, the higher the scores of their moral identity, online social support, and online altruistic behavior. In order to understand this relationship more deeply, the model constructed with moral identity and online social support as mediating variables shows that online interpersonal trust can have both a direct effect on online altruistic behavior and an indirect effect on online altruistic behavior through the mediating effects of moral identity and online social support. The specific mediating effects include two significant paths.

Firstly, the partial mediating role of online social support. Bandura’s social cognitive theory proposes that there is an interaction between the environment, individual factors, and behavior. In Internet research, researchers have attempted to introduce the theory to explain Internet use behavior, which acts as a social cognitive process (Larose et al., 2001; Larose and Eastin, 2004) in which the interaction between the Internet environment, the Internet users, and their behaviors collectively influences the manifestation and outcome of Internet use behavior. Online altruistic behavior is directly affected by the environmental factor of online social support. Specifically, the more social support an individual feels in the online environment, the more likely he or she is to go online to help others, thus generating altruistic behavior. Specifically, when individuals feel the care and support of others, they feel that they are an important part of the online community, and this sense of belonging makes them more willing to contribute to the community. At the same time, social support can also enhance the self-esteem level of individuals, so that they can face the challenges in the online world with more confidence and thus be more motivated to help others. In addition, online social support can also stimulate individuals’ gratitude (Liu, 2008). When individuals receive help from others, they develop an emotion of wanting to return the favor, and this emotion drives them to help more people in need. In the network environment, the gratitude emotion spreads very fast, and it can quickly stimulate more people’s altruistic behavior, thus forming a positive network atmosphere (Zhang et al., 2014).

Secondly, the chain mediating role of moral identity and online social support. Online interpersonal trust not only indirectly affects online altruistic behavior through online social support, but also influences online altruistic behavior through the role of moral identity on online social support. Moral identity is a kind of internalized noble ideal belief, reflecting the individual’s recognition of the importance of “being a moral person,” and moral identity is an important motivation to stimulate moral behavior. In addition, moral identity is also a kind of psychological needs. Research has shown that individuals with a high level of moral identity tend to compare the ideal moral self-image with the real moral self-image. When there is a gap between the two, this comparison triggers psychological pressure on the individual. And in order to alleviate this pressure, the individual is more likely to tend to adopt behaviors that are compatible with his or her own internal moral standards (Mulder and Aquino, 2013; Yang et al., 2015). Individuals who feel the awe of virtuous characters or behaviors will develop a virtuous aspiration and form a similar virtuous self-image, and those with a high level of moral self-identity will drive their behaviors to be consistent with their ideal image, thus prompting individuals to produce virtuous behaviors. That is, the importance of moral identity in self-concept is closely related to an individual’s moral cognitive ability (Aquino et al., 2009). Moreover, achieving self-consistency as a motivation for the internalization of moral identity requires that an individual’s perceptions and behaviors are consistent with his or her moral self-concept. As a result, individuals with high internalization will steadily display high moral sensitivity independent of contextual influences in order to maintain their self-consistency. Meanwhile, perceived social support, as a perception of self and interpersonal relationships (Lakey and Cassady, 1990), has an important influence on general self-concept (Tabbah et al., 2012). Individuals with a high degree of internalization have a moral self-concept that occupies an important place in the general self-concept (Aquino and Reed, 2002) and are susceptible to the influence of perceived social support. Individuals’ moral identity with the group may allow them to feel recognition and support from other members of the group, which in turn strengthens the feeling of social support. And from the perspective of moral motivation, high moral identifiers have a latent desire to produce ethical behaviors and feel more social support, which in turn creates a strong impetus for online altruistic behavior.

This suggests that in the future, colleges and universities should strengthen moral education and cultivate a high sense of moral identity among students, which can be achieved through curricula, social practices, and role models. Schools, social organizations, and enterprises can establish such online communities through online forums, social media platforms, and so on, encouraging members to support and collaborate with each other. This also can be achieved by strengthening online security education, establishing integrity mechanisms, and promoting a fair and just online environment and by giving out awards, organizing recognition activities, and providing scholarships to encourage more people to actively participate in online altruistic behaviors.

## 5 Conclusion

Through the research detailed above, we draw the following conclusions: (1) Moral identity, online social support, online interpersonal trust and online altruistic behavior were significantly positively correlated (*r* = 0.09–0.39, *p* < 0.01); (2) Online interpersonal trust has a significant positive predictive effect on moral identity, online social support and online altruistic behavior, that is, the higher the score of online interpersonal trust, the higher the score of moral identity, online social support and online altruistic behavior; (3) Online social support plays a single mediating role between online interpersonal trust and online altruistic behavior, accounting for 33.76% of the total effect, while moral identity and online social support play a chain mediating role between online interpersonal trust and online altruistic behavior, accounting for 2.23% of the total effect.

## Data Availability

The raw data supporting the conclusions of this article will be made available by the authors, without undue reservation.
